# Microbial Enhanced Heavy Oil Recovery by the Aid of Inhabitant Spore-Forming Bacteria: An Insight Review

**DOI:** 10.1155/2014/309159

**Published:** 2014-01-16

**Authors:** Biji Shibulal, Saif N. Al-Bahry, Yahya M. Al-Wahaibi, Abdulkader E. Elshafie, Ali S. Al-Bemani, Sanket J. Joshi

**Affiliations:** ^1^Department of Biology, College of Science, Sultan Qaboos University, 123 Muscat, Oman; ^2^Petroleum and Chemical Engineering Department, College of Engineering, Sultan Qaboos University, 123 Muscat, Oman; ^3^Central Analytical and Applied Research Unit, College of Science, Sultan Qaboos University, 123 Muscat, Oman

## Abstract

Crude oil is the major source of energy worldwide being exploited as a source of economy, including Oman. As the price of crude oil increases and crude oil reserves collapse, exploitation of oil resources in mature reservoirs is essential for meeting future energy demands. As conventional recovery methods currently used have become less efficient for the needs, there is a continuous demand of developing a new technology which helps in the upgradation of heavy crude oil. Microbial enhanced oil recovery (MEOR) is an important tertiary oil recovery method which is cost-effective and eco-friendly technology to drive the residual oil trapped in the reservoirs. The potential of microorganisms to degrade heavy crude oil to reduce viscosity is considered to be very effective in MEOR. Earlier studies of MEOR (1950s) were based on three broad areas: injection, dispersion, and propagation of microorganisms in petroleum reservoirs; selective degradation of oil components to improve flow characteristics; and production of metabolites by microorganisms and their effects. Since thermophilic spore-forming bacteria can thrive in very extreme conditions in oil reservoirs, they are the most suitable organisms for the purpose. This paper contains the review of work done with thermophilic spore-forming bacteria by different researchers.

## 1. Background

Oil productions have been experiencing decline in many parts of the world due to the oil field maturity, and example of such includes the major oil fields in the North Sea [1]. Another major factor which causes downgrade is the increasing energy demands due to global population growth and the difficulty in discovering new oil fields as an alternative to the exploited oil fields. Therefore, there is an urge to find out alternative technologies to increase oil recovery from existing oil fields around the world. It is a fact that fossil fuels will still remain the key source of energy, regardless of the gross investments in other energy sources such as biofuels, solar energy, and wind energy. Current global energy production from fossil fuels represents about 80–90% with oil and gas typifying about 60% [2]. Cossé [3] stated that during the process of oil production, between 30 and 40% of oil can be contributed by primary oil recovery, while additional 15–25% can be recovered by secondary methods such as water injection leaving behind about 35–55% of oil as residual oil in the reservoirs. The focus of many enhanced oil recovery technologies is this residual oil, and it amounts to about 2–4 trillion barrels [4] or about 67% of the total oil reserves [5]. For many oil companies, residual oil recovery is at present unavoidable, and so there is a perpetual hunt for a cheap and efficient technology which will raise the global oil production as well as the productive life of many oil fields. The recovery of this residual oil is accomplished by enhanced oil recovery (EOR) or tertiary recovery methods which are used in oil industry to increase the production of crude oil. Most common tertiary recovery methods include chemical flooding, miscible CO_2_ injection, and thermally enhanced oil recovery method which uses heat as a main source for the additional oil recovery [6]. Large quantities of residual oil in the depleted oil reservoirs could be regained by these EOR methods as the current primary and secondary extraction methods leave about two-thirds of the original oil in the reservoir. One of the potential EOR methods is microbial enhanced oil recovery (MEOR), which employs microorganisms to pull out the remaining oil from the reservoirs. Up to 50% of the residual oil can be extracted by this exceptionally low operating cost technology [7, 8]. The field trials of MEOR method project a chance to reverse the declining trend of oil production or at least to maintain a curve with a positive slope. This is achieved by the alteration of chemical and physical properties of reservoir rocks and crude oil by the microbial growth and metabolites produced [9]. MEOR can overcome the main hindrances of efficient oil recovery such as low reservoir permeability, high viscosity of the crude oil, and high oil-water interfacial tensions, which in turn result in high capillary forces retaining the oil within the reservoir rock [10].

## 2. The Reasons for Oil to Get Left Behind

The fundamental cause for leaving oil behind is economics. In general, the process of recovering oil from any conventional reservoir requires (a) a pathway which connects oil in the pore space of a reservoir to the surface and (b) sufficient energy in the reservoir to drive the oil to the surface. Lack of these requirements in the environment results in oil getting left behind. In this case, it is not economical to implement incremental development activities. In addition, all of the theoretically displaceable oil cannot be recovered, even if there is a pathway and adequate reservoir energy, due to the physics of fluid displacement in porous media [11].

## 3. Enhanced Oil Recovery

The residual crude oil in reservoirs is up to 67% of the total petroleum reserves in the world, which in turn represents the relative inefficiency of the primary and secondary production techniques. Extraction of this trapped oil can be achieved by injecting chemicals (polymers or surfactants), gases (carbon dioxide, hydrocarbons, or nitrogen), or steam into the reservoir. The chemicals used for EOR must be compatible with the physical and chemical environments of oil reservoirs. The varying permeability of petroleum reservoirs is also a major concern in EOR processes. When water is injected to displace the oil, it preferentially flows through areas of highest permeability and bypasses much of the oil [12]. Thus, the conventional EOR methods to recover the entrapped crude oil seem not to be very efficient.

## 4. Microbial Enhanced Oil Recovery (MEOR)

MEOR is a tertiary oil recovery technique. Recovering oil usually requires three stages. At the primary recovery only 12% to 15% of the oil in the well is recovered without the need to introduce other substances into the well. The oil well is then flooded with water or other substances to drive out an additional oil (15% to 20%) from the well which is known as the secondary recovery. Tertiary recovery is the last phase which is accomplished through several different methods, including MEOR, for the additional extraction of trapped oil from the well. In principle, the process of MEOR results in some beneficial effects such as formation of stable oil-water emulsions reduced interfacial tension and clogging the high permeable zones. In *in situ *MEOR method, bacteria inoculated with water in to the well will progress into high-permeability zones at first. Then at a later stage they will grow and occlude those zones due to their size and the negative charge on their cell surface. This scenario helps to increase the sweep efficiency, and thus a more efficient oil recovery can be achieved [11, 13].

Microorganisms can synthesize useful products by fermenting low-cost substrates or raw materials. Therefore, MEOR can substitute chemical enhanced oil recovery (CEOR), which is a very pricey technology. In MEOR, the chosen microbial strains are used to synthesize compounds analogous to those used in CEOR processes which are very expensive, to increase the recovery of oil from depleted and marginal reservoirs. Furthermore, microbial products are biodegradable and have low toxicity [7, 14, 15]. Microbial technologies are becoming approved universally as lucrative and eco-friendly approaches to improve oil production [16, 17].

## 5. MEOR Outcomes 

MEOR is based on two absolute justifications. Oil advancement through porous media is expedited by modifying the interfacial properties of the oil-water minerals. In such a system, microbial activity alters fluidity (viscosity reduction, miscible flooding); displacement efficiency (decrease of interfacial tension, increase of permeability); sweep efficiency (mobility control, selective plugging); and driving force (reservoir pressure).

The second principle is known as upgrading. In this case, the degradation of heavy oils into lighter ones occurs by microbial activity. Instead, it can also aid in the removal of sulphur from heavy oils as well as the removal of heavy metals.

Continuous research and successful applications affirm the fact that MEOR can be viewed as a potent technology [8, 22, 23] despite the existing disagreement by some groups [24]. However, successful MEOR field applications reported are specific for each well and published information to support economic advantages is lacking. MEOR is, therefore, considered as one of the promising future research areas with great preference as identified by the Oil and Gas in the 21st Century Task Force [24]. This is probably because MEOR is an alternate technology that may help in recovering the 377 billion barrels of oil that are unrecoverable by conventional technologies [8].

## 6. The Bygone Days of MEOR

It was Beckman in 1926 [25] who suggested for the first time that microbes could be used to recover oil from porous media. Between 1926 and 1940, not many studies were held on this topic. In the 1940s, Zobell [26] started a series of systematic laboratory findings which marked the beginning of a new era of petroleum microbiology research with application in oil recovery. According to Zobell the main mechanisms behind oil release from porous media are processes such as bacterial metabolites that break up inorganic carbonates; bacterial gases which reduce the viscosity of oil, thereby increasing its flow; surface-active substances or wetting agents produced by some bacteria; and the high affinity of bacteria for solids to crowd off the oil films, processes by which bacterial products (gases, acids, solvents, surface-active agents, and cell biomass) releasing oil from the sand pack columns in wet labs were patented by Zobell. Later Updegraff et al. repeated [27, 28] Zobell's experiments and patented [29] the process which is based on the bacterial byproducts produced from cheap substrates like molasses to assist the oil recovery. The first field test was carried out in the Lisbon field, Union County, AR [30]. Kuznetsov et al. [31] concluded that anaerobic bacteria present in the oil deposits can utilize oil to form gaseous products (CH_4_, H_2_, CO_2_, N_2_). Kuznetsov's work demonstrated the technology of microbial flora activation of reservoirs, later advanced by Ivanov et al. [32]. Extensive research on MEOR was conducted in the 1960s and 1970s, in Czechoslovakia, Hungary, and Poland [33–35]. The field trials were based on the injection of mixed anaerobic or facultative anaerobic bacteria (*Clostridium, Bacillus, Pseudomonas, Arthrobacterium, Micrococcus, Peptococcus, Mycobacterium*, etc.) selected on their ability to generate gases, acids, solvents, polymers, surfactants, and cell biomass. At the same time, another technology named as selective plugging recovery has been recognized as an important additional mechanism for improving the oil recovery from water floods. This is achieved by producing polysaccharide slime *in situ* by an injected microbial system based on molasses. Microbes producing biopolymers of xanthan or scleroglucan types as viscosifying agents were isolated, which greatly enhanced oil recovery [36–38]. The investigations during 1970–2000 have demonstrated the basic nature and existence of indigenous microbiota in oil reservoirs, as well as reservoir characteristics essential to a successful MEOR application. It was also proved that the cyclic microbial recovery (single well stimulation), microbial flooding recovery, and selective plugging recovery are very effective. The technology based on activation of stratal microbiota was successfully developed in former Soviet Union [32, 39]. It can be concluded that the petroleum crisis during 1970s led to substantial MEOR research and later became a scientifically identified EOR method, supported by research projects carried out all over the world in countries such as the USA, Canada, Australia, China, Russia, Romania, Poland, Hungary, Czech Republic, Great Britain, Germany, Norway, and Bulgaria. Many international meetings were periodically organized on the MEOR topic with the publication of proceedings carrying the advances in the knowledge and practice of MEOR techniques. It is important to recognize and acknowledge the role of the U.S. Department of Energy (DOE), which sponsored MEOR basic research and field trials, as well as periodically organizing international meetings. Several books on MEOR were also published [40–42]. Grula et al. [43] developed a microbial screening method to isolate an anaerobic *Clostridium* species that produced gases, acids, alcohols, and surfactants. But all those strains isolated showed intolerance to high salt concentrations (>7%) which remained as a major problem. Success of *in situ* MEOR processes depends upon isolating microorganisms that can survive and produce the desired metabolic products in reservoirs containing hydrocarbons and saline water. Continuous investigations were done on different microbial species such as *Clostridium *species, *Bacillus* species, and *Enterobacter* for better adaptation to reservoir conditions. By the end of the 1990s, MEOR was recognized as a scientific and interdisciplinary technique for the increase of oil recovery.

In 1995, a survey of 322 MEOR projects in the USA showed that 81% of the projects successfully increased oil production, and neither of them had shown reduced oil production [7]. Today, MEOR technologies are well suited for application, when there is a need for oil crisis at a rate of 3 to 4%/year. Since 1980, the abolition of stripper wells has increased to 175% [9], and accordingly, within 15–25 years, the USA could have access to less than 25% of its remaining oil resources. MEOR technologies were very slowly recognized by industry even though a long history of MEOR activity exists, due to the lack of published data especially in widely available journals, as well as too little cooperation between microbiologists, reservoirs engineers, geologists, economists, and owner operators.

## 7. Laboratory and Field MEOR Projects

Zobell [44] patented a process for the secondary oil recovery, using anaerobic, hydrocarbon-utilizing, and sulfate-reducing bacteria such as *Desulfovibrio *species *in situ*. He reported that the oil recovery mechanism was similar to *Clostridium*, where bacterial cells (and the hydrogenase enzyme system) produces the acids and ammonium hydroxide by using CO_2_, water and nitrates present in the reservoir, which helps to enhance the release of oil from reservoir rock when supplied with nutrients.

Various “agroindustrial carbohydrates based” substrates are proposed as a suitable “carbon source” for MEOR applications, like molasses [17, 45]. Updegraff and Wren [27] proposed that fermentative bacteria such as *Desulfovibrio *use nutrients such as molasses to produce large amounts of organic acids and carbon dioxide to enhance oil recovery in wet labs. The process was patented by them in spite of the major drawback of *Desulfovibrio *species producing hydrogen sulfide which is not suitable for MEOR processes. MEOR research team at Sultan Qaboos University, Oman, have reported isolation, identification, and bioproducts production by spore-forming *Bacilli *spp., and its potential role in enhancing oil recovery at laboratory scale [13, 17–21]. Bond [46] injected 5,000 gal of agar medium containing sand and *Desulfovibrio hydrocarbonoclasticus, *which is no longer a valid species into a sandstone reservoir at a depth of 3,000 ft. The well initially produced 15 bbl/day. After the inoculum injection, the well was shut in for 3 months for the bacterial growth and action. The well, when it started the production again, produced 25 bbl/day.

Hitzman [47] patented a process of injecting bacterial spores along with nutrients into a reservoir. The spores would germinate in the reservoir and enhance oil recovery from reservoir rock. A medium containing molasses and spores of *Clostridium roseum *was passed through the sand-packed column saturated with oil and showed about 30% increase in release of oil.

Patents by Hitzman [36, 47] used microorganisms that utilized injected polymers and the byproducts of CO_2_ floods, to produce products such as gases, acids, solvents, and surfactants for EOR. In polymer floods, the injected organisms feed on polymer that is adsorbed on the reservoir rock. In CO_2_ floods, the microbes feed on soluble compounds of carbon, nitrogen, and sulfur left behind by the CO_2_-crude oil slug. The process was demonstrated in sand-pack, but no core or field tests are reported.

Knapp et al. [48] reported the isolation of 22 microorganisms that produce biopolymers and emulsifiers. Among them, one strain could thrive at 10% salt concentrations, over a pH range of 4.6 to 9.0, at temperatures up to 50°C, in presence of crude oil. They demonstrated that glucose, ammonium sulfate, and potassium phosphate were easily transported through sandstone cores. Viable bacterial cells in aqueous solutions of 2% NaCl and 0.01% CaC1_2_ injected into these cores were not recovered in the effluent. The cores were inoculated with bacteria and nutrients such as glucose were added which resulted in a significant decrease in permeability. This could be because of the plugging of pores by the bacterial mass. The prominent bacteria indigenous to all of the cores treated were found as *Pseudomonas *sp., *Bacillus *sp., and Actinomycetes. A major problem in these experiments was the determination of the amount of plugging caused by injected bacteria and the amount by inhabitant ones. The problem existed even when cores were steam-sterilized and autoclaved. “Sterilization” of cores with chlorine dioxide helped to get rid of the problem, but the bacterial populations returned after 48 h incubation.

Johnson [49] studied 150 stripper wells in the USA that produced, on an average, 2 bbl/day, with no well head pressure. The reservoir porosities were 10 to 30%, depths 200 to 1,000 ft, with an average reservoir temperature of 38°C. In his study, he inoculated a mixed culture of *Bacillus *and *Clostridium *spp. (1 to 10 gal) with crude molasses and mineral salts as nutrients. Approximately 10 to 14 days were needed for the optimal growth of cells in the treated area of the reservoir. The results varied, but an average of 20 to 30% additional oil-in-place was recovered.

The preliminary field tests done by Petrogen, Inc., during 1977–88 in 24 wells with varying depths from 300 to 4,600 ft., demonstrated a pressure increase of 10 to 200 psi in 75% of the wells. Four wells doubled production for 6 months, and 12 increased production by 50% for 3 months. The average production increase was indicated as 42%; however, the final results remain to be reported [50]. Jack et al. [51] considered that emulsification of viscous crude oil *in situ *is not a feasible method for EOR since transporting the bacteria through the reservoir rock would face some difficulties. Yarbrough and Coty [30] reported a field test performed by them in 1954 in Arkansas, in which *Clostridium acetobutylicum* was injected along with a 2% solution of beet molasses in fresh water during a 6-month period. 70 days after starting the injection, freshwater breakthrough occurred at the production well. Fermentation products such as short-chain fatty acids, CO_2_, and traces of ethanol, 1-butanol, and acetone and sugars were found 80 to 90 days after the injection started. There was no increase in hydrogen content. Production of oil increased from 0.6 bbl/day to 2.1 bbl/day. Field test studies were not conducted.

The first MEOR project in the Rocky Mountains was started in 1983 [52]. An independent oil operator acquired three field service operations from Petroleum Bio-Resources Company. These were (a) a reservoir field conditioning system to avoid plugging; (b) use of a microorganism that produces gas and surfactant; and (c) use of a microorganism that produces a polysaccharide for mobility control. It was stated that production immediately doubled due to well stimulation and also increased oil recovery from 26 to 60 bbl/day, probably due to mobilization of oil by microorganisms, and water flooding was also noticed in other fields [53]. Bryant and Douglas [54] demonstrated the oil recovery efficiency of several different bacterial strains in Berea sandstone cores. They reported that additional 32% oil was recovered as compared to water flooding, and some spore-forming bacteria even showed 50–60% additional oil recovery. Berea sandstone core experiments showed that selected microbial strains could recover up to 72% of the heavy oil (API 14° and 17°) left after water flooding.

### 7.1. Field Tests

Kuznetsov [55] reported that bacteria were present in certain oil-gas-bearing strata in the Saratov and Buguruslan areas of the USSR in such numbers that large quantities of CO_2_ were generated (depth was approximately 3,300 ft). Certainly methane was also formed. In the later works, Kuznetsov et al. [31] introduced a mixed culture of aerobic and anaerobic bacteria with acid-hydrolyzed substances from peat and soils and shut in the well for 6 months, and after that the well was opened for production [50]. The rate of oil production rose from 275 to 300 bbl/day; however, 4 months later it had fallen to 270 bbl/day. Field tests were done by Dostálek and Spurny [33] in Czechoslovakia where they injected sulfate-reducing (*Desulfovibrio*) and hydrocarbon-utilizing (*Pseudomonas*) bacteria with nutrients (molasses). During six-month experiment period, the daily average oil production increased by nearly 7%. No further work has been reported since 1958. Heningen et al. [56] reported on two field tests performed in the Netherlands, in which they used *Betacoccus dextranicus* in a sucrose-molasses medium of 10% total sugar content and obtained a 30% increase in cumulative oil recovery. A mixed culture of slime-forming bacteria in 50% molasses was used in the subsequent field trial. The oil-to-water production ratio changed to 1 : 20 compared to 1 : 50 before the treatment.

In Hungary, to recover naphthenic crude, Jaranyi et al. [57] utilized a mixture of anaerobic thermophilic bacteria that fermented molasses. They also tried with raw sewage as an inoculum (100 L, along with 20 to 40,000 kg molasses) in their later trials (1969-70). The deepest reservoir was 8,200 ft, where the pressure was 228 atm and the temperature was 97°C. In 70% of the reservoirs tested, the introduced microbial populations showed positive results on overall oil recovery.

Karaskiewicz [58] conducted 18 field trials in Poland between 1961 and 1969. Microbial cultures were obtained from soil and water samples which were collected from the nearby areas of the oil fields and from sugar factory waters. The mixed culture includes the genera *Arthrobacter*, *Clostridium*, *Mycobacterium*, *Peptococcus*, and *Pseudomonas* grown in 10 L bottles with formation water plus 4% molasses, incubated at 32°C. The wells ranged in depth from 1,650 to 5,000 ft. The rate of additional oil recovery ranged from 20 to 200% of the original production rate. An additional supply of nutrients was proved to be a major factor for the increased oil recovery. Lazar [59] published an extensive review of MEOR work done in Romania during the last decade, in which he discussed three major areas in MEOR including (a) isolation of the bacterial population from the formation water of the reservoir; (b) adaptation of these microorganisms in wet lab for oil release; and (c) field testing of such adapted cultures. Seven wells were treated with microbial formulations, and he concluded that the bacterial population caused an increase of oil flow up to 200% for 1 to 5 years in 2 out of 7 reservoirs (the other five were unaffected), and much information about the ecology of the reservoir is needed before initiating any MEOR activity. A list of various reported successful MEOR applications at laboratory scale and field are listed in Table 1.

## 8. Heavy Oil

Heavy crude oil or extra heavy crude oil is a type of crude oil which does not flow easily. It is referred to as “heavy” because of its density or specific gravity, which is higher than that of light crude oil. Heavy crude oil has been defined as any liquid petroleum with API gravity less than 20°, which means its specific gravity is greater than 0.933. This type of oil forms due to the exposure of crude oil to bacteria [60]. Production, transportation, and refining of heavy crude oil are much difficult compared to light crude oil. The largest reserves of heavy oil in the world are located in the north of the Orinoco River in Venezuela (Energy Information Administration, 2001) the same amount as the conventional oil reserves of Saudi Arabia, but 30 or more countries are known to have such heavy crude oil reserves. Heavy crude oil is closely related to oil sands; the main difference is that oil sands generally do not flow at all. Canada has large reserves of oil sands, located north and northeast of Edmonton, Alberta. Physical properties that distinguish heavy crudes from lighter ones include higher viscosity and specific gravity, as well as heavier molecular composition. Extra heavy oil from the Orinoco region has a viscosity of over 10,000 centipoise and 10° API gravity. A diluent is added at regular distances in pipeline carrying heavy oil to increase the flow rate [61].

### 8.1. Field Tests

Heavy crude oil plays a major role in the economics of petroleum development. The heavy oil resources in the world are more than twice those of conventional light crude oil. In October 2009, the USGS updated the Orinoco tar sands (Venezuela) recoverable value to 513 billion barrels (8.16 × 1010 m^3^) (USGS. 11 January 2010), making this area the world's first recoverable oil deposit, ahead of Saudi Arabia and Canada [61]. The price of heavy crude oil slashes as compared to light oil due to increased refining costs and high sulphur content. The high viscosity and density also make production more difficult. On the other hand, large quantities of heavy crudes have been discovered in the Americas including Canada, Venezuela, and California. Another reason can be the relatively shallow depth of heavy oil fields (often less than 3000 feet) which contributes to lower production costs [62]. Special techniques are being developed for exploration and production of heavy oil.

### 8.2. Chemical Properties

Heavy oil contains asphaltenes and resins. It is “heavy” (dense and viscous) due to the high ratio of aromatics and naphthenes to paraffins (linear alkanes) and high amounts of NSOs (nitrogen, sulfur, oxygen, and heavy metals). The carbon chain in heavy oil has over 60 carbon atoms which results in a high boiling point and molecular weight. For example, the viscosity of Venezuela's Orinoco extra-heavy crude oil lies in the range of 1000–5000 cP (1–5 Pa*·*s), while Canadian extra-heavy crude has a viscosity in the range of 5000–10,000 cP (5–10 Pa*·*s), about the same as molasses, and higher (up to 100,000 cP or 100 Pa*·*s for the most viscous commercially exploitable deposits) [62]. A definition from the Chevron Phillips Chemical Company is as follows.

The “heaviness” of heavy oil is primarily the result of a relatively high proportion of a mixed bag of complex, high molecular weight, nonparaffinic compounds, and a low proportion of volatile, low molecular weight compounds. Heavy oils typically contain very little paraffin and may or may not contain high levels of asphaltenes.

## 9. Development of Heavy Oil Reserves in Oman

The first oil discovery in the Sultanate of Oman was accomplished in 1956, when City Services Company drilled Marmul-1 well. But the discovery was not considered as a commercial discovery because the oil found was heavy compared to oil discoveries in the Middle East at that time. In 1962, Petroleum Development of Oman (PDO) exploration activities ended up in achieving commercial discovery of oil in Yibal field, followed by discoveries in Natih and Fahud fields in 1963 and 1964, respectively. These discoveries marked the birth of Oman as an oil producing country. The result of these successes in discovering and production of oil inspired the Government to sign two new agreements to explore oil and gas in 1973 and another two in 1975 with other international oil companies. By 2009, the number of active oil fields reached 135. Over these days, Oman has been continuously applying efforts to improve the recovery of its oil reserves and has adopted EOR techniques on a large scale. These initiatives helped the Sultanate to increase their oil production capability to nearly 1 million barrels per day (bpd) from 714300 bpd averaged in 2008 [63]. This has also changed the outlook for its oil industry which is now estimated to have at least 40 years of life ahead of it [64]. Al-Ghubar South's discovery in 2009 was the most auspicious discovery for Oman. According to the Ministry of Oil and Gas, this discovery could add as much as 1 billion barrels to reserves. Two other convincing discoveries, including that in Malaan West and Taliah in the Lekhwair cluster in northwest Oman, were made which will stretch the baseline production in the future [65].

### 9.1. First Trials

The first export of Omani oil took place on July 27,1967. In the beginning, oil production increased steadily to 341000 barrels per day in 1975 and in 1984; the average daily production reached around 400000 barrels per day. Petroleum Development Oman (PDO)—the largest oilfield operator in Oman—started a series of EOR trials in 1986 due to low recovery of oil because of the complex geology of the reservoirs. The trials proved successful and Oman slowly started implementing EOR thereby boosting the production to a current level of nearly 900000 bpd. EOR projects result in 5–15 percent increment in reserves and PDO expects its EOR projects to contribute around 35 percent of its total production by 2020. So Oman is considered as a country which is pushing the limits of EOR technology [66].

#### 9.1.1. Miscible Gas Injection

Miscible gas injection involves pumping gas to oil wells. These gases that are being used for this purpose are often toxic which will dissolve in the oil and eventually lead to higher flow rates. This technique is currently at its operations in the Harweel oil field cluster [65].

#### 9.1.2. Steam Injection

Qarn Alam is the world's first full field EOR project and also the largest of its kind in the world. Thermally assisted gas oil gravity drainage (TAGOGD), a sophisticated method, is employed due to the characteristics of the fractured carbonate reservoir, as the oil is highly viscous and a very low percentage of recovery is feasible by conventional oil extraction method.

#### 9.1.3. Polymer Injection

Marmul field is located in south Oman. It is characterized by heavy viscous crude that is difficult to extract by traditional recovery methods. The reservoir has a viscosity of around 90 cP. The reservoir's sweep efficiency was modified by viscosifying the water with the addition of polyacrylamide polymers and then injected in the reservoir through polymer injection wells. The polymer flooding at Marmul field will increase a further recovery by 8000 bpd. By this technique, 10–15% increase in recovery levels from the Marmul reservoirs is predicted.

### 9.2. EOR Projects in Oman Oil Fields

Miscible gas injection has been applied in Harweel oil field which resulted in an additional production of 40,000 bbl/day. Thermal EOR methods are being deployed at Mukhaizna, Marmul, Amal-East, Amal-West, and Qarn Alam fields. Mukhaizna has already increased production to 50,000 bbl/day, and the other fields, Amal-East and Amal-West, are expected to raise the production to 23,000 bbl/day by 2018. Furthermore, the steam injection at Qarn Alam is supposed to enhance the production by 40,000 bbl/day by 2015. This is achieved by a novel process in which steam drains oil to lower producer wells. At projects such as Marmul, with its heavy oil reserves, injecting polymer fluid has seen to be more effective.

Other EOR projects include Karim cluster, a cluster of 18 oil fields flowing to the Nimr production facility, in which PDO is aiming to boost up the production. In Harweel cluster, PDO estimates approximately 40 percentage increase in the next five years. Also with Rima clusters, using EOR techniques, much gain is expected (US Energy Information Administration, 2012).

## 10. Role of Microbes in Biodegradation of Heavy Crude Oil

Degradation of oil is one of the most important parts of the MEOR by which the oil's viscosity and freezing point are reduced which in turn will increase the oil's flow *in situ*. Heavy oils are rich in gum and asphaltene, having characteristics such as freezing point, low flow ability, difficult oil recovery, and high recovery cost [67].

Microbe can improve the physical characteristics of heavy oil in two ways: (1) by degrading heavy oil fractions, thereby decreasing the average molecular weight of heavy oil; and (2) the byproducts of microbial metabolism, such as biological surface active substance, acid, and gas, which can reduce the viscosity of oil considerably. Gum and asphaltene present in heavy oil have high molecular weight and polarity; meanwhile they are one of the main factors making the oil recovery difficult [68]. Usually, microbe hardly degrades them [69]. Zhang et al. [70] separated a variety of microbes from environments rich in petroleum and done a series of experiments using mixed microbial consortia, which can effectively degrade heavy oil, even gum and asphaltene; these microbes act by lowering the viscosity and freezing point of heavy oil and thereby improving the physical and chemical characters of heavy oils.

In some cases, using microbial consortia with different properties (ability to degrade heavy oil fractions and biosurfactant production) thereby applying different mechanisms might have a desired effect for enhanced oil recovery [71]. There are a lot of microbes having the ability to degrade hydrocarbons by using them as carbon sources [72]. Interesting results for the microbial n-alkane degradation have been reported during the past decades [73–76]. Extensive studies have been made on strains of *Gordonia amicalis* which have shown to be a potent degrader of large n-alkanes under aerobic and anaerobic conditions [77]; many *Pseudomonas* species have the ability to degrade lighter hydrocarbons with carbon chain length C_12_–C_32_, and heavier hydrocarbons with carbon chain length of C_36_–C_40_ [78, 79]; and a thermophilic *Bacillus* strain that degrades only long-chain (C_15_–C_36_) hydrocarbons but not short-chain (C_8_–C_14_) n-alkanes [80] has also been reported. The ability of biosurfactant-producing indigenous *Bacillus* strains to degrade the higher fractions of crude oil and aid in the enhancement of its flow characteristics has also been studied for a petroleum reservoir in the Daqing Oilfield [81]. The MEOR team in the Sultan Qaboos University, Oman, found that a consortia of* Bacillus *strains form oil contaminated soil degraded heavy chain oil (C_50_–C_70_) to (C_11_–C_20_). Many microorganisms contain genes coding for the enzymes responsible for degrading petroleum hydrocarbons. Some microorganisms degrade alkanes (normal, branched, and cyclic paraffins), others aromatics, and others both paraffinic and aromatic hydrocarbons [82–84]. The most readily degraded alkanes are considered to be in in the range of C_10_ to C_26_, but low-molecular-weight aromatics, such as benzene, toluene, and xylene, which are considered as the toxic compounds found in petroleum, are also readily biodegraded by many marine microorganisms. As the complexity of the structures (those with branches and/or condensed ring structures) increases, it will be more resistant for biodegradation, which means only fewer microorganisms can degrade those structures and the biodegradation rates would be much lower than the rates for the simpler hydrocarbon structures found in petroleum. The higher the number of methyl-branched components or condensed aromatic rings, the slower the rates of biodegradation and the greater the probability of accumulating partially oxidized intermediary metabolites.

Petroleum contains numerous compounds of varying structural complexities. The residual mixture formed after petroleum biodegradation may resist further biodegradation. Crude oils are never completely degraded and always result in some complex residue which appears as a black tar containing a high proportion of asphaltic compounds. The toxicity and bioavailability of the residual mixture are very low as long as it does not coat and suffocate an area, thus becoming an inert environmental contaminant with no toxic effects on environment [60].

About 10% of the total bacterial population in hydrocarbon-contaminated marine environments is hydrocarbon-degrading bacterial populations [82]. The major metabolic pathways for hydrocarbon biodegradation have been elucidated [85]. The initial steps in the biodegradation of hydrocarbons by bacteria are the oxidation of the oil by oxygenases. Alkanes are subsequently converted to carboxylic acids that are further biodegraded via *β*-oxidation (the central metabolic pathway for the utilization of fatty acids from lipids, which results in the formation of acetate, enters into the tricarboxylic acid cycle). Generally aromatic hydrocarbon rings are hydroxylated to form diols, which are then eventually cleaved to form catechols which are subsequently degraded to intermediates of the tricarboxylic acid cycle. Interestingly, the intermediates resulting from bacterial action are with differing stereochemistry usually cis-diols, which are biologically inactive. With bacteria being the dominant hydrocarbon degraders in the marine environment, the products of aromatic hydrocarbons biodegradation will detoxify them and do not produce potential carcinogens. The complete biodegradation (mineralization) of hydrocarbons produces environmentally safe end products such as carbon dioxide and water, as well as cell biomass (largely protein) which will eventually enter into the food web.

## 11. Microbial Candidates Involved in Crude Oil Degradation

### 11.1. Thermophilic Spore-Forming Bacteria Involved in Biodegradation of Heavy Crude Oil for MEOR

Many varieties of microbes are identified and isolated from different petroleum reservoirs which comprise several ecological niches, including sulfate reducers [86–88], sulfur reducers [86], methanogen [88], fermentative bacteria [89, 90], manganese and iron reducers [91], and dibenzothiophene-degrading bacteria [92]. Although many bacteria are isolated from many reservoirs, those which can be applied to MEOR are fewer.

Many researchers have been engaged in studying thermophiles. It is reported that 140 species of 70 genera of thermophiles have been discovered from high temperature environments with wide applications [93]. In the Shengli oil field of East China, where extreme physical conditions exist with temperature ranging 60–90°C and depth of 1000–2000 m, most of the reservoirs are under EOR. This harsh environment seems to be unsuitable for microbial growth. But some thermophiles have been isolated which helps in EOR [86].

There are many kinds of *Bacillus*, which are distributed widely, but those which have application on crude oil recovery are very few [94, 95]. *B*. *subtilis* and *B*. *licheniformis* strains have been repeatedly isolated from many oil reservoirs as well as oil contaminated samples, thus confirming the adaptability of these species [17–19, 95–101]. The properties of *B*. *subtilis* have been reported in much literature [17–19, 96, 102–104], but the isolation and its action on crude oil have been scarcely reported [95].

It is recognized that the thermophiles possess enzymes which are more resistant to physical and chemical denaturation. Their faster growth rates also serve as another major advantage. Relative studies suggest that thermophilic hydrocarbon degraders of *Bacillus*, *Thermus*, *Thermococcus*, and *Thermotoga* species occurring in natural high-temperature or sulfur-rich environments are of special significance [105]. Wang et al. [106] isolated functional bacteria from high temperature petroleum reservoirs. Three thermophilic hydrocarbon-degrading bacteria, which belonged to *Bacillus* sp., *Geobacillus* sp., and *Petrobacter* sp., could tolerate 55°C in obligate anaerobic condition. These strains could utilize crude oil as carbon source with the degradation rate of 56.5%, 70.01%, and 31.78%, respectively, along with the viscosity reduction rate of 40%, 54.55%, and 29.09%, meanwhile the solidify points of crude oil were reduced by 3.7, 5.2, and 3.1°C.

Hao et al. [107] isolated a hydrocarbon-degrading bacterium, strain SB-1, from oil-contaminated soil samples collected at the Shengli oil field in east China. Based on 16S rDNA sequence, the strain was identified as *B. subtilis*. The bacteria degraded 39.33% of crude oil, 57.01% of the saturated fractions, 25.63% of the resins, and 12.15% of the aromatic fractions within 12 days. In addition, more than 50% of the alkanes were removed by the strain; the highest degradation rate was shown as 81.03% for C_36_–C_40_, and the lowest degradation rate being 51.47% for C_31_–C_35_. The results of this study concluded that *B. subtilis* SB-1 is a potent strain in degrading oil pollutants in soil.

Sanchez et al. [108] isolated thermophilic bacteria enriched from the formation waters of a Venezuelan oil field. The reservoir, located at Maracaibo Lake, has a temperature of 60–80°C and a pressure of 1,200–1,500 psi. The main fermentative byproducts were alcohols, short chain fatty acids, and gases when grown in media with industrial wastes as carbon source.

A strain of *B. stearothermophilus* (*Geobacillus*) was isolated from oil-contaminated Kuwaiti desert capable of growing on C_15_–C_17_ [109], and two strains of *G. jurassicus* were isolated from a high temperature petroleum reservoir capable of growing on C_6_–C_16_ [110]. *B. thermoleovorans* strain isolated from deep subterranean petroleum reservoirs was shown to degrade n-alkane up to C_23_ at 70°C [111]. Thermophilic, glucose-fermenting, strictly anaerobic, rod-shaped bacterium, Thermotoga hypogea sp. strain SEBR 6459T (T = type strain), was isolated from an African oil-producing well [112] and *T. elfii* strain SEBR 6459 by Ravot et. al. [113]. Al-Bahry et al. [18–21, 96] reported 33 genera and 58 species identified from Omani oil wells. All of the identified microbial genera were first reported in Oman, with *Caminicella sporogenes* for the first time reported from oil fields. Most of the identified microorganisms were found to be anaerobic, thermophilic, and halophilic and produced biogases, biosolvents, and biosurfactants as by-products, which may be potentially applicable in MEOR.

Various bioremediation and biodegradation agents are commercially available consisting of microbial cultures or microbial enzymes or both. The US Environmental Protection Agency National Contingency Plan released a product schedule report on August 2013 [114]. Also various laboratory screening reports are available for these commercial products [115].

## 12. Conclusions

Given the scarcity of the literature on thermophilic spore-forming bacteria involved in MEOR for crude oil biodegradation, there is a clear need for further laboratory research. While significant progress has been made, we still need to rigorously examine this mechanism of MEOR.

## Figures and Tables

**Table 1:** Successful laboratory and field MEOR applications [7, 13, 17–21].

Country	Biological systems used
USA	Pure or mixed cultures of *Bacillus, Clostridium, Pseudomonas,* and Gram-negative rods; mixed cultures of hydrocarbon degrading bacteria; mixed cultures of marine source bacteria; spore suspension of *Clostridium*; indigenous stratal microflora; slime-forming bacteria; ultramicrobacteria
Russia	Pure cultures of *C. tyrobutiricum; *bacteria mixed cultures; indigenous microflora of water injection and water formation; activated sludge bacteria; naturally occurring microbiota of industrial (food) wastes
China	Mixed enriched bacterial cultures of *Bacillus*, *Bacteroides, Eubacterium, Fusobacterium, Pseudomonas*; slime-forming bacteria: *Brevibacterium viscogenes, Corynebacterium gumiform, Xanthomonas campestris *
Australia	Ultramicrobacteria with surface active properties
Bulgaria	Indigenous oil-oxidizing bacteria from water injection and water formation
Canada	Pure culture of *Leuconostoc mesenteroides *
Former Czechoslovakia	Hydrocarbon oxidizing bacteria (predominant *Pseudomonas sp*.); sulfate-reducing bacteria
England	Naturally occurring anaerobic strain, high generator of acids; special starved bacteria, good producers of exopolymers
Former East Germany	Mixed cultures of thermophilic *Bacillus* and *Clostridium* from indigenous brine microflora
Hungary	Mixed sewage-sludge bacteria cultures (predominant: *Clostridium*, *Desulfovibrio, Pseudomonas*)
Norway	Nitrate-reducing bacteria naturally occurring in North Sea water
Oman	Autochthonous spore-forming bacteria from oil wells and oil contaminated soil
Poland	Mixed bacteria cultures (*Arthrobacter*, *Clostridium*, *Mycobacterium*, *Peptococcus, Pseudomonas*)
Romania	Adapted mixed enrichment cultures (predominant: *Bacillus*, *Clostridium*, *Pseudomonas*, and other Gram-negative rods)
Saudi Arabia	Adequate bacterial inoculum according to requirements of each technology
The Netherlands	Slime-forming bacteria (*Betacoccus dextranicus*)
Trinidad-Tobago	Facultative anaerobic bacteria high producers of gases
Venezuela	Adapted mixed enrichment cultures
